# Effect of estradiol on histopathology of brain in unilateral and bilateral ovariectomized rats

**DOI:** 10.6026/97320630019703

**Published:** 2023-06-30

**Authors:** Vaijayanthimala Ponnusamy, Kamalakannan Solaiyappan, Mydhili Govindarasu, Lavanya Prathap, Shyamaladevi Babu, Madhan Krishnan

**Affiliations:** Research scholar, Saveetha Medical College, Saveetha Institute of Medical and Technical Sciences (SIMATS), Chennai, Tamilnadu, India; Department of Anatomy,Sri Lalithambigai Medical College and hospital, Chennai, Tamilnadu, India; Department of Anatomy, Shri Sathya Sai Medical College and Research Institute, Sri Balaji Vidyapeeth (SBV) - Deemed to be University, Ammapettai, Nellikuppam, Chengalpet District, Tamil Nadu - 603108; Biomedical research unit and laboratory animal centre, Department of Anatomy, Saveetha Dental College and Hospitals, Saveetha Institute of Medical and Technical Sciences (SIMATS), Chennai, Tamilnadu, India; Department of Anatomy, Saveetha Dental College and Hospitals, Saveetha Institute of Medical and Technical Sciences (SIMATS), Chennai, Tamilnadu, India; Research, Chettinad Hospital and Research Institute, Chettinad Academy of Research and Education, Kelambakkam-603103, Tamil Nadu, India

**Keywords:** Ovariectomy, unilateral, bilateral, Brain, Histopathology, Estradiol therapy

## Abstract

Estradiol, a major form of estrogen, plays a crucial role in various aspects of brain function, including neuronal health, synaptic plasticity, and cognitive processes. Therefore, it is of interest to investigate the histopathological changes of the rat brain following estradiol treatment in unilateral and bilateral ovariectomized rats, a commonly used method to induce estrogen deficiency and study the consequences of hormonal changes. Two months old Wistar albino female rats were divided into five experimental groups, each consisting of 6 animals. Unilateral and bilateral ovariectomized rats were treated with estradiol (10 mg/Kg/b.wt) subcutaneously for 30 days. The histopathological analysis of brain revealed normal 5-6 compact layers of small pyramidal cells of CA3 region, most with vesicular nuclei in control rats andan irregular and disturbed thickness of the pyramidal cell layer in the CA3 region, suggesting neuronal loss in unilateral ovariectomized rats. Estradiol therapy to these rats showed dark hyperchromatic layers of pyramidal cells in the CA3 region, most of which displayed vesicular nuclei and less shrinkage. Bilateral ovariectomized rats without estradiol treatment showed irregular and disturbed thickness of the pyramidal cell layer in the CA3 region Similar to Group II, indicating neuronal loss and bilateral ovariectomized rats with estradiol treatment showed no significant changes. These findings highlight the potential role of estradiol in modulating the histopathological changes associated with ovariectomy. Further research is warranted to elucidate the underlying mechanisms through which estradiol exerts its effects on neuronal integrity and to explore potential therapeutic strategies for preventing or reversing these histological changes.

## Background:

Estradiol, a major form of estrogen, plays a crucial role in various aspects of brain function, including neuronal health, synaptic plasticity, and cognitive processes. The removal of ovaries through unilateral or bilateral ovariectomy is a commonly used method to induce estrogen deficiency and study the consequences of hormonal changes. The effect of estradiol treatment on the histopathology of the rat brain in unilateral and bilateral ovariectomized (OVX) rats has been an area of scientific interest and investigation [1].The histopathological evaluation of the rat brain following estradiol treatment in unilateral and bilateral ovariectomized rats provides valuable insights into the potential effects of estrogen on brain morphology and cellular integrity. This assessment involves examining various histological parameters, such as neuronal density, neuroinflammation markers, synaptic connectivity, and neurogenesis. Etrogen receptors are widely distributed throughout the brain, including regions implicated in cognitive function, such as the hippocampus, prefrontal cortex, and amygdala. These receptors mediate the effects of estradiol on neuronal structure and function. Estradiol can influence neuroplasticity by regulating synaptic proteins, modulating neurotransmitter systems, and promoting the release of neurotrophic factors, which are essential for neuronal survival and synaptic connectivity [2, 3].

Research studies have demonstrated that estradiol treatment can exert neuroprotective effects in the rat brain. It has been shown to reduce neuroinflammation, oxidative stress, and apoptosis, while enhancing neuronal survival, synaptic plasticity, and neurogenesis. These effects are thought to be mediated through various mechanisms, including the modulation of neurotransmitter systems, regulation of gene expression, and promotion of neurotrophic factors that contribute to the preservation of brain health and cognitive function [4, 5, 6]. Understanding the impact of estradiol treatment on the histopathology of the rat brain in unilateral and bilateral ovariectomized rats is crucial for unravelling the underlying mechanisms by which estrogen influences brain structure and function. This knowledge can have important implications for the development of therapeutic strategies targeting neurodegenerative disorders, cognitive impairment, and mood disturbances associated with hormonal changes.

## Materials and Methods:

## Animals:

Two months old Wistar albino female rats were kept under a 12/12 h reversed light/dark cycle and in compliance with the NIH Guide for Care and Use of Animals. Lights were turned off at 8:00 h. During maze testing, they were kept at 80-85% of their usual body weight by limiting their food consumption. Each week, five grams of weight rise were permitted for growth.

## Ovariectomy procedure:

Rats were given general anaesthesia, 60 mg/kg of ketamine hydrochloride intramuscularly, and 50 mg/kg of sodium pentobarbital intraperitoneally, before the ovaries were removed unilaterally and bilaterally through midline abdominal incisions. The uterine horns and arteries were tied off 0.5-1 cm in front of the ovary. Adipose tissue was cut and ligated before the residual tissue was returned to the abdominal cavity. The wounds were sutured using a monofilament suture. A preventive dosage of 4000 IU benzathine penicillin G, was given intramuscularly after the procedure, and the animal was left to awake from anaesthesia [7]. After recovery from anaesthesia the animal was immediately returned to its cage. The animal was kept in a heated cage (25-27 °C) for at least two hours following surgery. The animals were kept apart for the first few days after surgery. The cage was regularly cleaned while the animal was recovering. A subcutaneous injection of 5 mg of Rimadyl per kg of body weight in saline was administered to the animals twenty-four hours following surgery for postoperative pain treatment [8].

## Hormonal treatment:

Rats underwent ovariectomy and were divided into 5 study groups (n= 6, each). 10 milligrams of 17b-estradiol-3-benzoate were diluted in 100 millilitres of sesame oil and administered to two experimental groups (unilateral OVX and bilateral OVX). Control groups were given 100 ml of sesame oil twice, separated by 24 hours. For morphological and molecular research, the rats from the groups were then slaughtered 30 days [9] later, as appropriate.

## Experimental design:

Animals were divided into five groups and each consisting of 6 animals.Group I- Normal rats; Group II- Unilateral ovariectomized rats without estrdiol treatment; Group III- Unilateral ovariectomized rats with subcutaneousestrdiol therapy (10 mg/Kg/b.wt) for 30 days; Group IV- Bilateral ovariectomized rats without estrdiol treatment. Group V: Bilateral ovariectomized rats with subcutaneous estrdiol treatment (10 mg/Kg/b.wt) for 30 days.

## Histopathology:

A section of brain was embedded in 10% neutral buffered formalin, sectioned, and stained with hematoxylin and eosin dye for histopathological analysis [10]. The semi-thin sections (0.5-1 microns) were then cut using a LKB ultra-microtome, stained with toluidine blue, examined with a light microscope fitted with a digital camera, and photographed at a 400x magnification.

## Results and Discussion:

**Figure 1:**
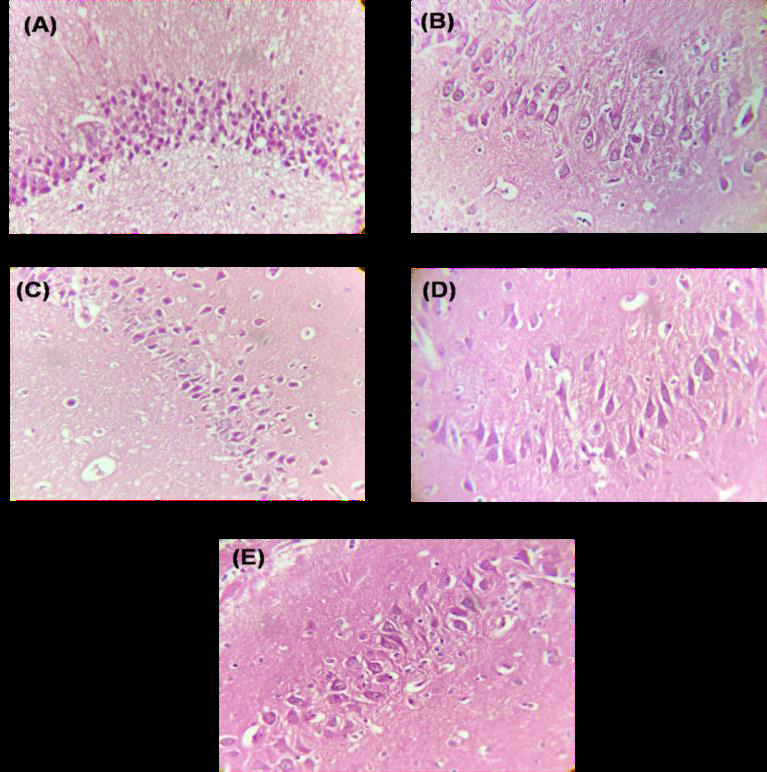
Effect of Estradiol treatment on Histopathology of rat brain in Unilateral and Bilateral Ovariectomized Rats: (A) Normal control group showed 5-6 compact layers of small pyramidal cells of CA3 region, most with vesicular nuclei. (B) Group II (Unilateral ovariectomized rats without estradiol treatment) exhibited apoptotic neurons with dystrophic changes like shrunken hyperchromatic, irregular with chromatolysis, and abnormal Nissl granule distribution, as well as irregular, disturbed thickness of the pyramidal cell layer in the CA3 region. (C) Group III (Unilateral ovariectomized rats receiving estradiol therapy) exhibited layers of pyramidal cells with dark hyperchromaticity in the CA3 region, the majority of which had vesicular nuclei and less shrinkage. (D)Group IV (bilateral ovariectomized rats without estradiol treatment) exhibited apoptotic neurons with dystrophic changes like shrunken hyperchromatic, irregular with chromatolysis, and abnormal Nissl granule distribution. It also displayed an irregular, disturbed thickness of the pyramidal cell layer in the CA3 region. (E) Group V (bilateral ovariectomized rats with estrdiol treatment) displayed shrinking pyramidal cell layers, pyknotic nuclei, and granular cytoplasm.

Over the past three decades, the effects of the oestrogen 17b-estradiol (E2) on the hippocampus have been attracting more and more attention. A wide range of hippocampal morphology and physiology in females is regulated by E2, including synaptic plasticity, neurogenesis, cell signalling, gene transcription, epigenetic processes, CA1 dendritic spine density, and protein translation [11]. The histopathological analysis of the rat brain in the study investigating the effect of estradiol treatment on unilateral and bilateral ovariectomized rats revealed distinct histological changes in the CA3 region of the hippocampus. In the normal control group, the histopathological examination showed 5-6 compact layers of small pyramidal cells in the CA3 region, with most cells exhibiting vesicular nuclei (Figure 1). This normal histological pattern indicates the typical morphology and organization of neurons in this brain region. Group II consisted of unilateral ovariectomized rats without estradiol treatment. The histopathological analysis revealed an irregular and disturbed thickness of the pyramidal cell layer in the CA3 region, suggesting neuronal loss. Additionally, the presence of apoptotic neurons with dystrophic changes, such as shrunken hyperchromatic nuclei, irregular nuclear shape, chromatolysis (dispersion of Nissl granules), and abnormal Nissl granule distribution, further indicates cellular dysfunction and degenerative changes [12, 13]. It is may be due to the less production of estradiol which is involved in various biological process as mention above, within the body as a result of unilateral ovariectomy [11].

The histopathological examination of unilateral ovariectomized rats with estradiol therapy showed dark hyperchromatic layers of pyramidal cells in the CA3 region, most of which displayed vesicular nuclei and less shrinkage compared to Group II. This finding suggests a protective effect of estradiol treatment on the histology of the CA3 region, potentially preventing or attenuating neuronal loss and degenerative changes. It has been elucidated in previous studies that oestrogen has the ability to protect against the oxidative activity [14], β-amyloid precursor protein [15], neuroinflammation [16, 17, 18], which are the pathophysiological factors associated with ischemic neuropathology that coincides with the preset results

Bilateral ovariectomized rats without estradiol treatment showed irregular and disturbed thickness of the pyramidal cell layer in the CA3 region Similar to Group II, indicating neuronal loss. The presence of apoptotic neurons with dystrophic changes, including shrunken hyperchromatic nuclei, irregular nuclear shape, chromatolysis, and abnormal Nissl granule distribution, further confirms the degenerative changes observed in this group. Considering, bilateral ovariectomized rats with estradiol treatment showed a pyramidal cell layers with shrinkage, pyknotic nuclei (condensed and fragmented nuclei), and granular cytoplasm. These histopathological features indicate adverse effects of both bilateral ovariectomy and estradiol treatment on the histology of the CA3 region. It has been reported in previous study, that in both rats and mice, bilateral ovariectomy decreases the density of the CA1 spine. This effect can be reversed within 30 minutes with a systemic injection of E2 or a dorsal hippocampal infusion of E2. The rapid activation of extracellular signal-regulated kinase (ERK) and mammalian target of rapamycin (mTOR) cell signalling in the dorsal hippocampus is required for these effects, which can be mediated by ERa, ERb, or GPER.The phosphoinositide 3-kinase (PI3K) and ERK signalling kinases act upstream to activate the mTOR pathway, which in turn causes dendrites to rapidly translate local proteins that result in spine remodelling and additionally essential for male rodents' hippocampal synaptic plasticity and memory development, as well as for the consolidation of E2-induced memories in mice with ovariectomies [11]. The observed histopathological changes in Groups II, IV, and V suggest that unilateral and bilateral ovariectomy without estradiol treatment leads to neuronal loss and degenerative changes in the CA3 region of the hippocampus. Estradiol therapy in unilateral ovariectomized rats (Group III) appeared to provide some protection against these histological alterations. However, in bilateral ovariectomized rats (Group V), estradiol treatment did not prevent the adverse effects on the histology of the CA3 region.

## Conclusion:

These findings highlight the potential role of estradiol in modulating the histopathological changes associated with ovariectomy. Further research is warranted to elucidate the underlying mechanisms through which estradiol exerts its effects on neuronal integrity and to explore potential therapeutic strategies for preventing or reversing these histological changes.
